# Local Value Chain Models of Healthy Food Access: A Qualitative Study of Two Approaches

**DOI:** 10.3390/nu13114145

**Published:** 2021-11-19

**Authors:** Kathleen Krzyzanowski Guerra, Andrew S. Hanks, Zoë T. Plakias, Susie Huser, Tom Redfern, Jennifer A. Garner

**Affiliations:** 1 John Glenn College of Public Affairs, The Ohio State University, Columbus, OH 43210, USA; 2 College of Education and Human Ecology, The Ohio State University, Columbus, OH 43210, USA; hanks.46@osu.edu; 3 Department of Agricultural, Environmental and Development Economics, College of Food, Agricultural and Environmental Sciences, The Ohio State University, Columbus, OH 43210, USA; plakias.2@osu.edu; 4 Community Food Initiatives, Athens, OH 45701, USA; donationstation@communityfoodinitiatives.org; 5 Rural Action, The Plains, OH 45780, USA; tomr@ruralaction.org; 6 School of Health and Rehabilitation Sciences, College of Medicine, The Ohio State University, Columbus, OH 43210, USA

**Keywords:** local food, food value chains, community food security, Appalachia, qualitative research

## Abstract

Food value chains are increasingly recognized as more equitable alternatives to traditional supply chains and may represent a novel mechanism to achieve health equity at the local level. Country Fresh Stops (CFS) and Donation Station (DS) are two complementary programs that are part of a more robust value chain designed to support local agriculture in Appalachia Ohio. As the first study of these programs in the peer-reviewed literature, the objectives were to identify factors that facilitate or hinder the implementation of these two local value chain models of healthy food access and to identify the perceived impacts from the perspective of the sites implementing them. In-depth, semi-structured interviews were conducted with CFS (*n* = 7) and DS (*n* = 10) site representatives in January 2020. Template analysis was used to identify themes through *a priori* and inductive codes. Participants identified two primary facilitators: support from partner organizations and on-site program stewardship. Produce (and program) seasonality and mitigating food waste were the most cited challenges. Despite challenges, both CFS and DS sites perceive the models to be successful efforts for supporting the local economy, achieving organizational missions, and providing consumers with greater access to locally grown produce. These innovative programs demonstrate good feasibility, but long-term sustainability and impacts on other key stakeholders merit further investigation.

## 1. Introduction

Increasing community-wide access to healthy foods—particularly fresh produce—is an oft-cited intermediate objective in efforts to reduce diet and health disparities [1,2,3]. One mechanism for doing so is via unique value chain models of equitable food access. Value chains have emerged as an alternative to the traditional supply chain, with distinct focus on benefitting participants equitably, creating shared value for community stakeholders, and producing positive social impacts [4]. The concept of shared value is underpinned by the assumption that markets have the potential to generate positive externalities as well as private benefits [5] and intersects with the tenets of social enterprise. That is, business practices do not focus solely on profit-seeking behaviors, such as enhancing firm competitiveness and economic growth, but also contribute to wider societal, communal, and environmental conditions [4,5,6,7].

Food value chains (FVCs), specifically, are framed in the literature as supporting participants in agri-food supply chains that may lack power relative to larger firms [4,8]. This includes small- and mid-scale farmers, ranchers, and regionally-based processors, distributors, and retailers [5,7,8]. Notably, FVCs—sometimes referred to as values-based supply chains or values-based food supply chains—can offer outlets suitable for small and mid-scale producers to distribute differentiated food products (e.g., products marketed as “locally grown”) through strategic alliances forged with supply chain partners [9,10]. Shared mission and agreed upon values are particularly important factors in food value chains that effectively enhance product value for consumers [4]. FVCs often include a combination of for-profit, nonprofit, and government entities to ensure greater provision of benefits generally underprovided by free markets.

FVCs are becoming more prevalent alongside a growing consumer preference to eat fresh, local, and organic produce [8,11,12,13]. Concurrently, local food assistance programming has expanded as the government increasingly looks to nonprofit organizations as partners in reducing food insecurity nationally [14,15,16,17,18]. Given the salience of FVCs in both for-profit and nonprofit sectors and the well-established influence of the food environment on diet-related behaviors, FVCs may represent a novel and understudied mechanism by which to achieve health equity at the local scale. Though there are different conceptualizations of “local” food, with some conflation of geospatial proximity with relational proximity, the concept of local remains a driving force behind innovative, value-based food supply chains [10,19]. For this work, we adhere to the definition of local food as that which is “raised, produced, and processed in the locality or region where the final product is marketed,” [12] (p. 2). Local FVCs focused on increasing access to fresh foods may help to address the impacts of food insecurity [20]. This article explores the factors that facilitate and impede the implementation of two programs, Country Fresh Stops (CFS) and Donation Station (DS), that are part of a local value chain in the Appalachian region of rural Ohio. These programs aim to address persistent diet and health inequities observed in the region by facilitating community food security—“a situation in which all community residents obtain a safe, culturally acceptable, nutritionally adequate diet through a sustainable food system that maximizes community self-reliance and social justice” [21] (p. 37).

### 1.1. Country Fresh Stops and Donation Station Programs

CFS and DS are two complementary programs for increasing healthy food access and facilitating community food security in Athens County and the greater Appalachian Ohio region. These programs are part of a larger value chain that facilitates food procurement and access and are inclusive of economic, social, environmental, and equity goals [4,22]. Early conversations with partnering nonprofit organizations highlighted the unique localized nature of these programs. Rather than rely on a national network of in-kind food assistance providers, such as Feeding America, that may be accompanied by administrative burdens [23], both programs reflect the intentional employment of local infrastructure and resources [24] to address local issues.

CFS is a market-based program (i.e., situated within the retail or for-profit food environment) that was created in 2010 and continues to be managed by a local nonprofit organization, Rural Action (RA). The final consumers must pay prices for fresh produce as set by retailers through this program. CFS-affiliated sites work with RA to source locally grown produce and offer it for sale on a consistent basis, reflecting the programmatic goal of making fresh produce more widely accessible for sale at retail markets across the region. Participating locations include traditional store venues (mainly small, locally owned stores) or weekly “pop-up” markets at established organizations (e.g., healthcare settings) [25]. These sites set their own purchasing budgets, and RA takes on the responsibility of procuring a satisfactory bundle of produce for each site accordingly. RA staff purchase the produce from local farmers at the Chesterhill Produce Auction, a social enterprise owned and operated by RA at which a robust network of regional producers sells their goods twice weekly throughout the growing season [26]. A key feature of this auction is that Amish producers are important sellers and play a pivotal role in sustaining the auction. Amish producers—members of an Amish community in and around the Chesterhill area of Morgan County, Ohio—use the Chesterhill Produce Auction as a primary market for their produce. While most of the producers who sell through the auction are non-Amish, the majority of the produce sold through the auction comes from Amish producers. There are several produce auctions in Ohio, which aggregate multiple farmers to create a destination for produce [27]. Wholesale produce auctions allow purchasers to negotiate prices through competitive bidding, while also supporting farmers in obtaining high prices relative to traditional farmers market models [28]. RA purchases produce from the auction on a weekly basis and then delivers it immediately to participating CFS locations, along with related signage and point-of-purchase materials.

DS is an assistance-based program (i.e., situated within the nonprofit food environment) that was created in 2007 and continues to be managed by a different local nonprofit organization, Community Food Initiatives (CFI). Final consumers receive fresh produce for free through this program. With monetary donations, CFI purchases seasonal produce on a weekly basis throughout the growing season from regional farmers at the Chesterhill Produce Auction. CFI then distributes these goods to local food access partners (hereafter referred to as “DS-affiliated sites”) who serve food insecure clientele [29]. CFI also accepts donations of produce from local growers, those shopping at the Chesterhill Produce Auction, and farmer markets where the CFI is present. DS-affiliated sites—a revolving mix of regional food pantries and prepared meal sites—receive the produce for free at structured weekly CFI-hosted pick-up events. It is subsequently offered to DS site clientele for free as part of pantry bundles or incorporation into provided meals.

**Figure 1:**
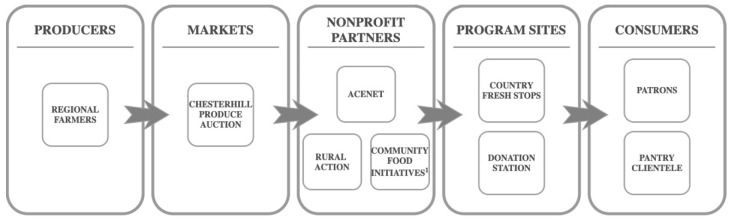
Country Fresh Stops and Donation Station: local value chain models for healthy food access. ^1^ CFI(Community Food Initiatives) supplements produce purchased from the Chesterhill Produce Auction with produce sold by regional farmers at the Athens Farmers Market. CFI also accepts donations of produce.

The Appalachian Center for Economic Networks (ACEnet) provides critical support to the nonprofits who manage CFS and DS, as well as the sites that implement the programs. This support includes staff assistance, warehouse space for produce storage and distribution, and grant-writing expertise that enables the funding necessary for program continuation. CFS and DS are enabled via the collaborative logistical support of RA, CFI, and ACEnet. Together with ACEnet, RA and CFI represent the Appalachian Accessible Food Network, a coalition aimed at strengthening the local food value chain [30]. Figure 1 illustrates the local value chain within which these programmatic models operate.

There is a significant body of literature that explores the persistence of food insecurity despite the availability of both federal food assistance programs and more localized interventions, such as food pantries [17,31,32,33,34]. For example, studies have found that food assistance programs that are run nationally but implemented locally, such as Feeding America’s weekend backpack program, are effective in providing supplemental foods, but may not adequately address nutrition gaps [31]; transportation and proximity to food assistance program offices are related to community food security [17], which has several implications for rural and remote areas; and food pantries may not always be able to offer clientele access to high-quality, nutritionally dense, or culturally-appropriate options [32]. Studies also suggest that charitable food assistance (i.e., nonprofit food assistance) use serves as a mechanism for low-income households to mitigate food insecurity, particularly in times when federal food assistance benefits wane [33]. In terms of market-based approaches to healthy food access, research indicates that improving neighborhood access to healthy food retailers does not necessarily lead to improved dietary outcomes [34]. Within this vast literature, though, there is a dearth of scholarship on the potential of local FVCs to increase healthy food access, especially in areas with high rates of food insecurity [35,36].

While RA, CFI, and ACEnet collectively partner with dozens of sites in Southeast Ohio to implement CFS and DS, and have done so for over a decade, neither program has been systematically evaluated in the peer-reviewed literature. This study fills this gap in the literature and contributes an understanding of these innovative, complementary programs and their respective implementation. Identifying and understanding the factors that facilitate and hinder the successful implementation—and potential impacts—of these programs and their shared value chain has practical, political, and academic value. These programs have the potential to improve outlets for and access to locally grown produce; help mitigate food security, diet, and health disparities in Appalachia and other culturally similar regions; and may be models for broader dissemination, given their alignment with national, bi-partisan produce promotion initiatives (e.g., the Gus Schumacher Nutrition Incentive Program) [37].

### 1.2. Objectives

There were two objectives of this research. First, we aimed to identify factors that help (facilitate) or impede (challenge) implementation of two complementary local value chain models of healthy food access, one *market-based* and the other *assistance-based*, with attention to similarities and differences in such factors across programmatic models. Second, we aimed to identify the perceived impacts of these models—for sites, clientele, and the broader community—from the perspective of the sites implementing them. As part of a larger, multi-stage, mixed-method evaluation effort, this qualitative study aimed both to generate transferable insights regarding local FVC implementation in a rural region and to inform subsequent strands of the research [38]. As municipal policy makers seek to strengthen local food systems and foster economic development throughout the rural United States, a foundational understanding of local FVC implementation, with implications for model sustainability, is essential to well-informed decision making and resource allocation.

## 2. Materials and Methods

### 2.1. Study Area

Athens County, nestled in southeast Ohio, is one of 32 counties in the state that are within the Appalachian region. Athens is a nonmetropolitan county, with approximately 43% of the population living in rural areas [39,40]. It is designated as a county of persistent poverty and low employment [41], the only Ohio county with both persistent poverty and persistent child poverty [42], and one of five Ohio counties that the Appalachian Regional Commission (ARC) categorizes as economically distressed (2020) [43]. The distressed designation of Athens presents an interesting case relative to the larger Ohio area; recent estimates suggest that 27% of the county’s population lives in poverty and 19% are experiencing food insecurity [44,45], relative to 14% in poverty and 13% experiencing food insecurity state-wide [44,46]. A related concern in Appalachia is the high rate of obesity and obesity-related chronic illnesses [1,47,48].

While these circumstances demonstrate an area navigating multi-layered hardship, Athens is known for its innovative local food system [49]. There are over 700 farms and almost 99,000 acres of dedicated farmland in Athens County [50]. In line with trends in the larger Appalachian region, agriculturally oriented social and regional entrepreneurship have been strategically undertaken in the Athens area [51], with the local FVC models explored here an exemplar of such efforts. The county is also home to Ohio University, an undergraduate- and graduate-degree-granting institution with over 28,000 students [52].

### 2.2. Methodology

**Figure 2:**
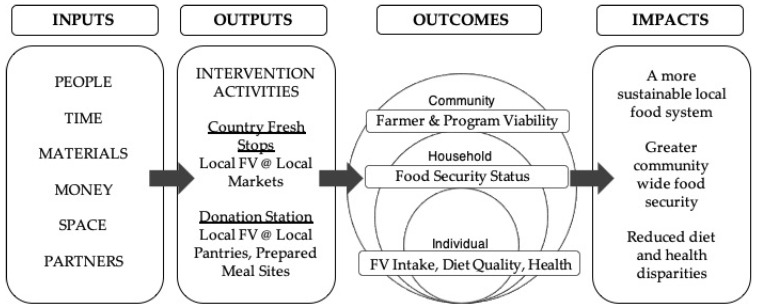
Programmatic logic model.

This qualitative study relied on a pragmatic philosophy of knowledge in that it assumes knowledge can be useful for both theoretical and practical purposes [53]. Pragmatism underscores the functional characteristics of knowledge; in other words, knowledge need not be confined to the bounds of inquiry but, rather, should be linked to useful action and not to clarifying “universal truths” [53,54]. In line with this pragmatic philosophy, the study team embraced the ideals of community-based participatory research [55]; the original research question—(how) are CFS and DS working for the community?—was a function of a multi-hour meeting between the PI and community-based partners during which a variety of mutually beneficial projects were discussed and a research priority established. That meeting instigated the grant through which this work, and subsequent aspects of the broader, sequential evaluation, was funded. All partners, including interdisciplinary scholars and community-based experts, worked collaboratively to specify the study aims and methods. Initially, this involved an intensive, in-person session to establish a shared framework of actors and processes applicable to the two programmatic models, which was then used to generate a unified, albeit general, logic model of how—and toward what end—the programs were expected to work (Figure 2). The logic model served as a programmatic theory of change to inform subsequent data collection [56].

Interviews with consumer-facing stakeholders were considered essential to establishing a more detailed understanding of program implementation and enhancing the logic model’s nuance. Semi-structured interview guides that reflected logic model constructs were co-created by the partners for use with CFS and DS site representatives (see Appendix A, Table A1 and Table A2 for full interview guides). While tailored to the program of interest, both guides included sections of open-ended questions on (1) site context (inputs), (2) program participation processes and perceptions (activities), (3) site clientele (audience), and (4) perceived program impacts for the site, clientele, and the community (outcomes and impacts). The Ohio State University approved the study protocol (#2020E0001) and deemed it exempt from full board review.

### 2.3. Participant Sampling

Seventeen semi-structured, in-depth qualitative interviews were conducted with a purposive sample of representatives from Country Fresh Stops sites (*n* = 7 of 8 active sites in 2019) and Donation Station sites (*n* = 10 of approximately 50 affiliated sites in 2019). A purposeful maximum variation sampling strategy allowed us to gather in-depth insights from a variety of participants across site locations who have first-hand knowledge of the realities faced in implementing the two programs of interest on a day-to-day basis [55,57,58,59,60]. Purposeful maximum variation is a useful sampling strategy to discover both program variation and commonalities [54]. Following Gioia et al., an important underlying assumption of this research was that the people within organizations of interest are knowledgeable agents with the ability to describe their intentions and actions and that of the broader organization [61]. For this research, all sites affiliated with each programmatic model were contacted to assess interest, and representatives across different sites who serve as the end implementers of the models were offered the opportunity to participate in an interview. As end implementers, these individuals are not employed by the nonprofit partners who manage CFS and DS. Rather, they are owners, employees, or volunteers at locations (e.g., local businesses, food pantries) where regional consumers—the end user and target beneficiary of these local FVC models—are served. A representative of RA and CFI recruited potential CFS and DS representatives, respectively, via email using their existing list of program contacts. To maximize the sample, partners followed up once or twice with non-responding representatives.

### 2.4. Data Collection

Willing representatives were scheduled for a 1 h, in-person interview conducted in January 2020. The objectives, requirements, and risks/benefits of the study were clearly outlined, and informed written consent, including permission to audio-record the interview, was obtained from each interviewee. To promote consistency, all interviews were conducted by the study’s principal investigator (J.A.G) who has formal training in and experience teaching qualitative research at the graduate level and nearly 10 years of experience with qualitative data collection and analysis. Probing techniques were employed, as needed, to support the exhaustive exploration of site-level context and implementation processes and experiences. In accordance with institutional incentive policies, all interviewees received a gift card incentive: $30 for Kroger. Kroger was identified by community-based partners as the most acceptable outlet from among the available options. Interview audio was transcribed verbatim for coding.

### 2.5. Data Analysis

Transcripts were coded in NVivo qualitative analysis software (version 12, QSR International Pty Ltd., Doncaster, Victoria, Australia 2018) according to the tenants of template analysis. Template analysis affords the researcher a systematic, yet flexible, approach to thematic analysis; namely, it allows for both inductive and deductive coding processes through the use a partial *a priori* coding framework that is useful for research questions that seek to compare perspectives across sets of stakeholders and approaching such datasets with some consistency [62,63]. The initial *a priori* codes—largely descriptive and categorical codes drawn from the interview guides and related program logic model—were integrated with inductively generated codes to guide the analysis [62,63].

Prior to coding and creating the initial template, the research team familiarized themselves with the data; this required listening to and reading interview transcripts concurrently, several times over. Preliminary coding was conducted by three individuals from the research team, with each person coding a subset of interview transcripts and identifying items of interest and relevance to the research question [62,64]. Preliminary codes were brought together, and the team clustered codes into meaningful groups in a hierarchal fashion [65], with parent codes and related child subcodes. The initial template was revisited and refined through iterative rounds of consensus coding on a large subset of the interviews, with two coders coming to an agreement on a final template and detailed code definitions. A primary coder proceeded to code all interviews using the final template. Consensus checks were conducted in two ways: the first with a small subset of interviews between the two coders who finalized the template and the second using an independent coder who is familiar with the research project but was not part of the team that created the initial nor final template [66]. The final template was then used to interpret the data.

## 3. Results

**Figure 3:**
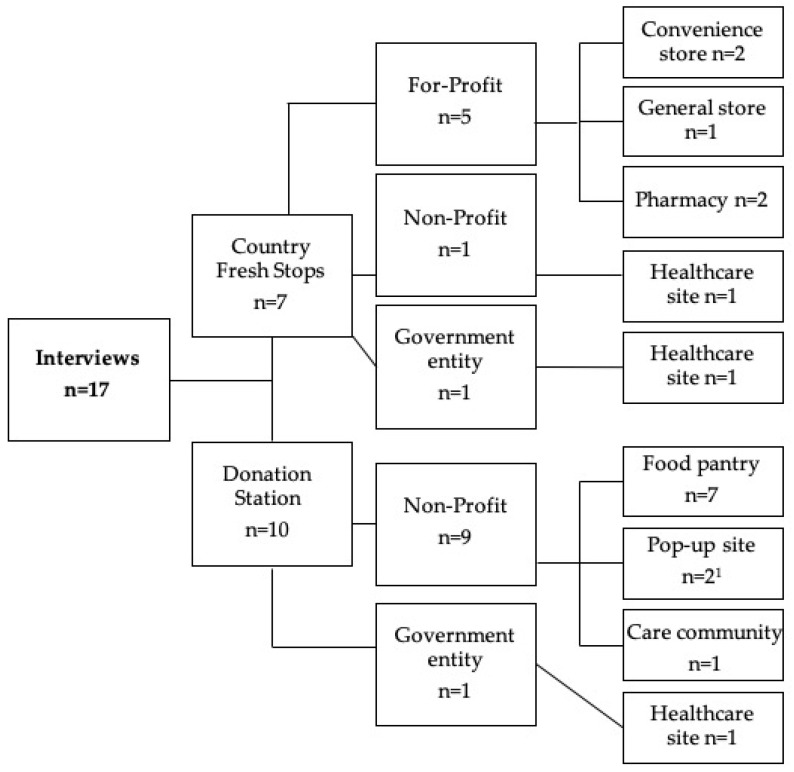
Site affiliate categorizations. ^1^ Pop-up sites included a school and a library.

Seventeen participants from seven CFS sites and ten DS sites were interviewed. Among the CFS sites represented, two were pharmacies, two were convenience stores, two were healthcare sites, and one was a general store. Among the DS sites, seven were food pantries, two were pop-up sites (one at a school, one at a library), one was a care community, and one was a healthcare site. Figure 3 depicts site affiliate categorizations.

Major themes across and within each local FVC model were organized into three sections according to the research question: program facilitators, program challenges, and perceived impacts. Additional illustrative quotes are included in Appendix B, Table A3.

### 3.1. Program Facilitators

Two major cross-model themes related to factors facilitating program implementation emerged: support from coordinating nonprofit organizations (partner support) and program stewardship.

#### 3.1.1. Partner Support

As the intermediaries facilitating the purchase, distribution, and supply of locally grown produce, the three partnering nonprofit organizations—ACEnet, CFI, and RA—provide crucial support to the sites implementing each program. Site representatives perceived the support provided by one or more of the partners as key to the successful incorporation of fresh fruits and vegetables into their established business or organizational practices.

RA provided CFS-affiliated sites with signage, marketing materials, storage for produce (e.g., refrigerated displays, display stands), and delivery services. Additional support is provided via help navigating produce orders from the Chesterhill Produce Auction and volunteer power, where applicable; pop-up markets at healthcare centers, in particular, benefit from RA-employed AmeriCorps volunteers who assist with RA produce deliveries and pop-up site operations. Many representatives also discussed the value of partner-supported cooking demonstrations, recipe cards, and taste-testing events for patrons. While such technical and logistical assistance was discussed and appreciated, it was the overall responsiveness of RA and ACEnet staff and the collaborative nature of the partnership that emerged as being critical to site satisfaction with the program:

*“I felt that they listened very well … So, I have not been disappointed at all. I mean, if I told them, well, I really don’t need this. Then they would change it for next week. Or that, these came but they really were not in good shape. They would credit it. So, I felt that they were open … I mean, they sit down, they meet with you, they talk to you.”*

Similar to the support that RA offered CFS-sites, CFI shared recipes with and conducted cooking demonstrations at DS-affiliated sites. Like CFS site representatives, DS site representatives discussed the general responsiveness of CFI and their efforts to maintain open communication. CFI staff also went above and beyond facilitation of the core produce distribution events by contacting sites when extra produce was available for pick-up between such events. A common sentiment expressed by DS site representatives was the critical role that CFI plays in providing a mechanism—which may not be available otherwise—for organizations focused on food access to offer fresh produce to their clientele:

*“They* [Donation Station staff] *do a really great job and I’m really glad that we have them, that we’re in partnership with them because I don’t know what we would do [for fresh produce] if we didn’t have Donation Station.”*

#### 3.1.2. Program Stewardship

Program stewardship—conceptualized as *site-level* processes or activities aimed at facilitating or promoting the success of CFS or DS—encompassed various actions taken by site staff to support each program’s implementation and foster its impact at their particular location. Stewardship took many different forms at CFS sites. Staff leveraged social media for marketing (letting patrons know when produce came in and what was available), engaged in real-time produce promotion through face-to-face patron interactions, and invested time into creating attractive produce displays and connecting patrons with relevant recipes:

*“I suggested to the Country Fresh Stop* [partners] *that they provide recipes for people because we were ending up giving people recipes … Some people didn’t know what to do with zucchinis or some people wouldn’t know what to do without kohlrabi or something. So, we came up with the idea that they could provide one recipe and people could just take a picture with their phone or write it down. And that seemed to help a lot.”*

In contrast to CFS stewardship activities centered around the marketing of available produce, program stewardship at DS sites included collaboration with other community organizations working to mitigate food insecurity (e.g., rapidly rerouting any produce leftover after a pantry event); volunteering extra time to attend CFI-hosted produce distributions; and incurring personal costs (e.g., gas and mileage associated with transit to and from the distributions). Though there was general agreement about the worthiness of such stewardship as a means to support healthy food access in their communities, the significance of time and costs invested into the success of DS and their larger operations was echoed by many site representatives:

*“Everyone that we have come in contact with, the people at the Donation Station, the people at the Auction, are so giving that how could we not? And we can help other people. The cost, yeah, it costs some money, but not that much, you know? We’re not overly wealthy but we can do that much. That’s just a little bit, but it’s more time. It’s a lot of time …”*

### 3.2. Program Challenges

Both the assistance-based (DS) and market-based (CFS) local FVC models were largely perceived by the interviewed site representatives as valuable endeavors. However, these site representatives also voiced challenges that hindered program implementation and potential impact. Challenges discussed most frequently across interviews were food waste and produce seasonality. DS sites also faced a unique challenge related to CFI’s produce distribution process.

#### 3.2.1. Food Waste

As for-profit entities paying for the produce with intent to sell it to customers and recoup these costs, CFS-affiliated sites had a relatively low tolerance for food waste. Site staff actively mitigated food waste and its associated impact on their profit by using unsold produce for their food service operations (e.g., using it in deli products sold to patrons). Those without such operations or produce-applicable menu items often donated it to local entities that serve food insecure individuals (e.g., DS-affiliated pantries), promoting use of the produce but preventing them from recouping the cost. Food waste seemed to cause a greater challenge for sites new to the program and still familiarizing themselves with ordering, storage, and patron demand:

*“Yeah, just getting used to the whole program. It was not known, really, not well advertised maybe. And we probably threw more away or gave it away than we sold, but then it kind of turned around … Well, that was probably when we were actually doing our own orders, so it was really hard to gauge … It made it a lot easier when* [Rural Action] *just brought a variety because they knew what’s in season, what’s selling, what’s not. And I had less waste.”*

For this reason, CFS site representatives were eager to learn more about proper produce storage and strategies for extending the shelf life of fresh foods, and some actively sought help from the organizational partners accordingly.

DS sites perceived food waste as less of a challenge relative to CFS sites, with patron demand being more reliable by nature of the produce being free. On occasions when produce was left undistributed at DS-affiliated sites, representatives discussed incorporating it into free meal services, composting it, donating rotten or past-prime produce to local farmers for animal feed, and rerouting excess produce to other food pantries in the area:

*“We have two different pantries at the end of our distribution to clients that take the leftovers … So if we have some produce [leftover] which is typically not a lot … Whoever has the pantry soonest takes those items … So nothing, nothing gets wasted.”*

#### 3.2.2. Seasonality

The variety and abundance of locally grown produce is not consistent year-round, causing confusion and concern for CFS sites. Such seasonality was perceived as negatively impacting patron satisfaction with and willingness to purchase produce from CFS-affiliated sites:

*“Sometimes we would get a whole bunch of green peppers and onions, and then there wouldn’t be a lot of variety. So, it would be kind of whatever fruit, whatever produce was in season, which I think is common. But I think patients were somewhat frustrated, that they were like, ‘Well if I go to Krogers and purchase this, then I can get a lot more variety, so I’m not going to shop here’.”*

In contrast to CFS, which only functions during the growing season, DS is a year-round program. However, like CFS, seasonality presented a concern for DS site representatives, given patron expectations for consistency in programming:

*“Wintertime is tough because we’re not getting as much produce. Then, especially as people start relying on that or are more accustomed to getting that produce every week or so from their community health worker. Then, when that dies off, they’re like, ‘Oh where are my veggies?’ But CFI does a really good job at like doing what they can … It’s just not as abundant.”*

(Site that partners with a social service agency to deliver food to homebound patrons)

#### 3.2.3. Donation Station Pick-Up Processes

A challenge of unique relevance to the DS site experience was the produce pick-up process and environment at CFI-hosted produce distribution events. The DS model, unlike CFS, does not include complementary transportation of and refrigerated storage for the produce. Affiliated sites benefit from access to free produce, but detailed the time and resources committed to attending the distribution events and their strategic approach to selecting produce for their site, keeping in mind the time that would pass before their next pantry or meal service day and their site’s storage capacities. Moreover, the sites discussed navigating an increasingly crowded and chaotic environment at the produce distributions. Growing demand for CFI-provided produce complicated the shared mission among those in attendance, and a competitive rather than collegial aura came to characterize the process as more sites in the region sought this valuable resource:

*“Well, this summer it kind of came to a head and we expressed our concerns about the aggression of those* [fellow site affiliates] *who were coming for food … We were very confused as to whether there was a limitation on the amounts and there seemed to be rather limited order or no order or very little structure.”*

### 3.3. Perceived Impacts

Representatives from both CFS- and DS-affiliated sites largely perceive the programs having positive impacts. Three major themes emerged related to perceived impacts: supporting local, patron satisfaction, and mission advancement.

#### 3.3.1. Supporting Local

Across the sample, “supporting local”—that is, purchasing from regional farmers, expanding food access options for the community, and helping to preserve the local economy—emerged as a primary theme related to both models’ perceived benefits for sites, their patrons, and the broader community. Site representatives recognized the opportunity to bring local produce to patrons who may not otherwise have access and framed its value as twofold: increasing patron access to healthy food and supporting local producers:

*“I don’t think we would have moved forward, at least if it wasn’t locally sourced … The produce auction is such a massive operation that we were pretty confident that we would have a good quantity and variety of produce on a weekly basis … I think that’s primarily the reason we wanted to move forward was that economic development piece.”*

(CFS-affiliated site)

#### 3.3.2. Patron Satisfaction

Representatives perceived that patron satisfaction was directly or indirectly related to their participation as a CFS- or a DS-affiliated site. CFS-affiliated sites perceived patron satisfaction related to increased accessibility fresh produce, and locally sourced fresh produce in particular. For DS-affiliated sites, participation in the local value chain model of produce procurement facilitated a sense of food system equity in which such produce was made available to patrons who may not have had access otherwise:

*“And so just to have that option of fresh, nutritious, local produce is lovely. And people connect and are grateful, really grateful when they have that option. And they’re so impressed when we get it. And we brag about it. It’s a fresh local produce. Not that … I mean who could say, no I don’t want apples from Washington? But it’s just really special that it’s southeastern Ohio feeding southeastern Ohio.”*

(DS-affiliated site)

#### 3.3.3. Mission Advancement

The programs also represented an opportunity for sites to advance their own organizational missions. Site missions for CFS-affiliated locations included providing stable employment for locals, serving the community, contributing to a strengthened regional economy, and fulfilling customer needs and preferences. Participating in the CFS model enabled them to support the local economy via their purchase of produce from regional farmers; provided a unique procurement opportunity, especially as small businesses without the means (or geographic proximity) to source produce via traditional supply chains; and resulted in a marketable expansion of their product mix—one that had perceived potential to drive customer satisfaction, traffic, sales, and long-term business viability:

*“I think it helps because it’s providing something that in prior years was never even an option for a convenience store. I would call it a rare opportunity because of us having a produce auction nearby, and able to get produce from that auction that is grown locally, to me is just outstanding.”*

(CFS-affiliated site)

For CFS-affiliated sites in healthcare settings, the model enabled sites to address patient health more holistically and to play a greater role in advancing community well-being.

DS-affiliated sites shared a common mission of serving under-resourced households and mitigating food insecurity, and participation in the DS model enabled them to enhance their services through the provision of fresh produce to their patrons. Like the healthcare-oriented CFS sites, DS sites recognized the important role of produce in promoting patron well-being, particularly those burdened with or at greater risk for diet-related chronic diseases:

*“We have a goal to improve the quality of life of the people that we’re interacting with. We’re interacting with people who are in poverty or in food insecurity and, like he mentioned earlier, all of our food is donated … And so the Donation Station has been a valuable resource as far as getting whole produce and then people have choice … So it’s helped economically for our organization as well as just being a source of really high-quality produce.”*

(DS-affiliated site)

While the importance of supporting local (i.e., supporting the local economy and producers) by sourcing locally was shared by affiliates of both models, the degree to which it motivated program participation differed. For CFS sites, the local orientation of the model was a marketable and motivating factor in their initial interest and continued affiliation. For DS sites, supporting local farmers was a plus, but the primary motivation and benefit was the free access to fresh, high-quality produce; its origination was of relatively less consequence.

## 4. Discussion

Our semi-structured interviews with CFS and DS site representatives yielded rich contextual insights into the implementation and perceived impacts of market-based and assistance-based local value chain models of healthy food access. As the formative phase of a multi-stage research project, this study provides the grounded knowledge essential for beginning to evaluate the sustainability and outcomes of two potentially scalable models for community-based healthy food access. As the first study of these programmatic models in the peer-reviewed literature, our analysis contributes to the scholarship on local FVC models of healthy food access and advances broader conversations regarding market- and assistance-based approaches for promoting community food security.

We identify common program facilitators, challenges, and perceived impacts across these distinct, but complementary, models. The related themes that emerged raise important questions about program implementation, sustainability, impact, and potential scalability. More specifically, the interplay of organizational-level investments (partner support) and site-level practices (program stewardship) that support implementation are juxtaposed with programmatic challenges (seasonality, food waste, and associated costs of participation), despite site-level motivation to continue program participation.

The two primary facilitators of these models were partner support and program stewardship. Partner support occurs at both organizational and individual levels, while program stewardship encompasses individual behaviors at the site level that facilitate program implementation. The significance of resources—financial, in-kind, and otherwise—that RA, CFI, and ACEnet invest into the CFS and DS programs should not be understated. The findings suggest that without the considerable support of partner organizations and the program stewardship undertaken to facilitate implementation, these programs would likely not be financially feasible nor sustainable (conceptualized herein as the ability to continually uphold and fulfill commitments to clients, patrons, and the community in which they operate) [67].

The individual- and organizational-level factors reflected in the concepts of program stewardship and partner support that emerged from this analysis are in line with the notion that constraints may arise due to the significant focus placed on non-financial returns of investment (e.g., fulfilling social missions) rather than financial returns [68]. As their respective social missions remain paramount for the nonprofit partners and are used to justify their ongoing program support, there is a need to further examine the sustainability of the models over the long term. This is of particular importance for nonprofits in resource-scarce environments and that face competing stakeholder demands [23].

Program stewardship is not as well discussed in the literature on value chains and social enterprise; however, employee buy-in has been attributed to resources expended for program implementation [69]. That is, the perceived value that employees or volunteers place on a program and its associated goals may connect to how much time and effort is placed on facilitating implementation. Though program stewardship emerged as a meaningful factor facilitating CFS and DS program implementation in the short term, questions remain about the impacts of such stewardship in the long term, particularly on long-term sustainability.

Implementing and managing a novel program or intervention tends not to be without some challenges [69,70]. Seasonality and food waste are common issues for programs that rely on local supply [70,71,72]. Food waste presents greater challenges for the market-based program (CFS) than the assistance-based program (DS). As discussed, participating CFS locations allocate funding toward the purchase of produce from the Chesterhill Produce Auction, which is then re-sold to patrons. Though some of the site representatives framed CFS as a budget-neutral venture, losing money due to unsold produce or spoilage presents concerns for long-term program sustainability. Conversely, DS sites receive produce at no cost, and participants indicated their sites have strong networks within the region, which allows for the quick re-routing of leftover produce. Existing research indicates that community networks serve a critical role in local food systems and localized interventions, particularly in rural areas [73,74]. CFS representatives did not discuss having similar networks to rely upon for the purpose of mitigating food waste; however, even if they are able to re-route unsold produce, they still risk not being able to recoup the costs of buying produce. This begs important questions about the relative feasibility of the models and whether local value chain models for healthy food access are more conducive to assistance-based approaches wherein there are existing networks to mitigate food waste and less emphasis on profitability. More practically, challenges around food waste and seasonality present programmatic opportunities to incorporate staff and volunteer trainings around storing fresh produce, generate signage, and market produce as local and in season.

A unique challenge for the assistance-based approach that emerged from the interviews was the competitive produce distribution environment. Competition arises amidst limited resources and the non-exclusionary model that CFI has created to avoid prioritizing certain food access partners over others. However, this competition may conflict with the community values that were discussed in relation to the programmatic facilitators of partner support and program stewardship, as well as the type of trust and cooperation framed as essential to effective value chains [4,7]. While the literature supports that fear of competition in local food systems is an issue for producers [49] and tightening of resource sharing among nonprofits may occur due to an increasingly resource-scarce environment [23], there is a need to investigate further the different areas where competition arises in local value chain models and what the implications may be for long-term program sustainability.

In terms of practice, findings reinforced concerns of which the coordinating nonprofit organizations were well aware. Specifically, concerns of a competitive CFI distribution environment prompted CFI to implement important changes to distribution events. At the time of data collection, CFI employed a free choice distribution method. However, in March 2020, CFI sought to alleviate competition at the point of distribution by re-structuring its process, including raising and allocating funds to commission farmers to grow food during times of lower supply, engaging DS-affiliated partners more frequently to assess their needs and satisfaction, and staggered distribution pick-ups to allow DS-affiliated sites the time and space to interact with DS staff. Importantly, these changes have required a considerable increase in DS resources (time, effort, and personnel) expended to ensure a more cooperative environment.

Perceived impacts of supporting local producers and community, mission alignment, and patron satisfaction emerged across both models. A sense of pride associated with supporting the local producers, community self-sufficiency, framing of local food systems as a mechanism to support regional economic development, and the social desirability of participating in the local food economy are findings aligned with the existing literature [49,74,75,76,77]. Mission alignment was more important to DS sites relative to CFS sites, particularly as CFS sites focused more attention on the cost of participating in the program. For sites that lost money or had perceived losses associated with CFS, questions arose about the desirability and feasibility of continued program participation. Conversely, for DS sites, mission alignment was incredibly important and emerged in different ways across sites. Thematic across many interviews with DS site representatives were social missions that included supporting the health and well-being of the Athens County population, improving the quality of life, and strengthening (or providing) a sense of community. These sentiments are similar to those expressed in other studies of local food systems, where building and maintaining community is a motivating factor for local producers to participate in localized food access interventions [22,74].

### 4.1. Strengths and Study Limitations

Limitations of the study include a relatively small sample, though saturation was still achieved, and the limited geographic context in which the studied models operate. Even so, the discussed alignment between study findings and the existing literature suggest that these models are subject to similar considerations as would be appropriate to other localized models of healthy food access across other contexts. Finally, the findings offer insights from the perspective of only one set of stakeholders: site representatives. While this enhances analytical coherency, a need remains to consider the perspectives of other key stakeholders, including farmers, nonprofit partners, and target consumers.

The rigor of this inquiry and credibility of the findings were enhanced through the development of a detailed, consensus codebook via an iterative, group process of transcript review, codebook application, and codebook revision [78,79]; the sharing, discussion, and “member checking” of preliminary findings with community partners and stakeholders via interim reports and team calls [80]; and the ongoing, collaborative research partnership composed of interdisciplinary scholars and community practitioners to facilitate interpretive rigor [79].

### 4.2. Implications for Research and Policy

The local food value chain programs explored in this analysis present innovative and potentially scalable models for promoting community food security and addressing persistent diet and health inequities. Local food initiatives have recently secured permanent funding in the recurring Farm Bill, suggesting that evaluating the relative sustainability of two innovative local programs is particularly timely [81,82]. Further, research on local food security programming is of particular importance for policy makers as the United States continues to navigate the COVID-19 pandemic and its food system impacts. Innovative local value chain models are an important focus of inquiry in the context of increased federal interest in supporting food security, food supply chains, farmers of color, and rural communities [83,84,85,86,87]. Questions remain about the two models’ respective efficiency and measurable impacts on the dietary patterns and health outcomes of community members; providing a systematic qualitative evaluation of such local value chain programs serves as an essential first step toward evidence-based policy and funding allocation.

Program stewardship emerged as an important facilitator for CFS and DS program implementation in the short term; however, questions concerning long-term sustainability and scalability arise in terms of the direct and indirect costs of such stewardship and its implications for the ongoing feasibility of CFS and DS. This gray zone introduces interesting elements for subsequent study and practice considerations moving forward. Specifically, there should be inquiry into how short-term program facilitators may become long-term barriers and how the models can be optimized to maximize site buy-in, while minimizing (or at least managing more strategically) site resource expenditures in terms of both time and direct costs. Related to sustainability, future research might examine whether these models are scalable for other regions with similar demographic characteristics.

Given that competition related to pick-up processes was overwhelmingly discussed by DS site representatives as a challenge, future research might also examine how “coopetition,” or simultaneous cooperation and competition [88], occurs between community-based organizations collaborating to achieve programmatic goals. Finally, future research might examine how (if at all) consumers align with site representative perceptions of CFS and DS programs related to sustainability, increased access to fresh produce, and the importance of supporting local in consumer purchasing decisions. Understanding site representative perceptions and values relative to consumer preferences will allow for a far more nuanced understanding of patron satisfaction, as well as perceived impacts by those that use CFS or DS as a means of sourcing produce.

## Data Availability

The data presented in this study are available on request from the corresponding authors. The data are not publicly available due to the nature of qualitative data, which contain sensitive information and pose a high risk of revealing the identity of study participants.

## Appendix A

**Table A1:** Country Fresh Stop Site Interview Guide.

Topic	Questions
Store Characteristics	What is the name of your store (or operation)? What is the store’s (or operation’s) address? Tell me a little about (store name). What are the main products sold? What foods do you sell? For how many years has this operation been in business? What are the operating hours? How many individuals are employed here? On average, how many customers do you serve in a day? Does it vary? How much produce (in lb) do you typically sell? What portion (%) of your sales is represented by fresh produce? Do you accept federal food assistance benefits (e.g., WIC, SNAP)? What is your organization’s mission? What are some challenges that you face in pursuing this mission? (If active site) How does your role as a Country Fresh Stop help you to achieve that mission?
Program Perceptions and Participation	Did your site serve as a Country Fresh Stop in 2019? (If yes) For how many years have you done so? (If no) For how many years did you do so? In what year did you stop? Tell me about your experience as a Country Fresh Stop. Why did you originally choose to be a Country Fresh Stop? What appealed to you about the program? What has gone/went well? What are/were the challenges of being a Country Fresh Stop for your establishment? Is there anything that you would like to see changed about the program? (For current CFS sites) Are there any factors that may impact your willingness to be a Country Fresh Stop moving forward? (For prior CFS sites) Why did your store stop participating as a Country Fresh Stop? What resources or assistance would/would have help(ed) you to be a successful Country Fresh Stop? How satisfied are/were you with the training, resources, and support provided to you as a Country Fresh Stop partner? How familiar are you with the produce auction from which most Country Fresh Stop produce is sourced (the Chesterhill Produce Auction)? How important was the source of the produce to you when considering your decision to participate as a Country Fresh Stop? Are you aware of how local farmer communities benefit from your participation as a Country Fresh Stop? (If needed, explain the provenance of the produce purchased at auction and distributed to consumers.) Would this knowledge of benefitting local farmers affect how your organization operates at all? Why/why not?
Produce Inventory	Did you sell produce prior to becoming a Country Fresh Stop? (If yes) Did you offer any locally sourced produce? (If yes) Have you continued sourcing produce from anyone other than Rural Action? (If yes) Who/how/how often? What % is from Rural Action? (If no) Why did you stop sourcing from them? How often did/do you receive produce replenishments for Country Fresh Stops? How many pounds of produce did/do you typically get per shipment from Rural Action? What factors dictate how frequently you request produce? What factors dictate how much produce you request (e.g., storage, demand)? Does the amount vary? What factors drive this variance (e.g., changing demand, changing capacity, etc.)? Do you request certain types of produce or let Rural Action dictate what you get? (If request) What types of produce do you request most frequently? *Show pictures and check off mentioned produce.* (If request) How consistently are you able to get the produce that you request? (If request) What factors drive your request for certain types of produce? Is there any produce that patrons prefer consistently over others? Which one(s)? How much does the type of produce that you sell vary from week to week? Are there any types of produce that patrons opt not to take? Which ones? How much, if any, produce are you unable to sell on a weekly basis? (Ask for %) (If any unsold) What do you do with produce that you are unable to sell?
Pricing and Cost	How do you go about setting your produce budget for Rural Action? What is your typical produce budget? Does it vary week to week? How much does the quantity of produce that you receive vary week to week? (If it varies) What factors drive this variance (e.g., varying weekly budget, varying value of produce purchased on a fixed budget, varying demand for different quantity/mix of items)? Do you add a mark-up to the produce sourced from Rural Action? (If no) Why not? (If yes) What is your typical mark-up? How do you set this? Is the produce sourced from Rural Action labeled or marketed as being part of Country Fresh Stops? Is the Donation Station produce marketed any differently from other produce—such as canned or frozen—that is offered to patrons? (If any marketing) What costs do you incur in marketing your Country Fresh Stop produce to patrons? (Probes: cost of materials? Of staff time?) What strategies do you use to promote produce selection and consumption among your patrons (e.g., recipe tastings, recipe cards)? Do you have (additional) strategies for encouraging patrons to select the produce sourced from Rural Action (e.g., display placement)?
Clientele	Describe the clientele of your operation. Who is your target customer? Do you see the same clientele frequently? To your best estimate, what is the average value of produce per client transaction? What other items, if any, do your customers tend to purchase *with* produce? Is there a time of the week or time of the day that you observe more produce purchases? What kinds of feedback have you received from patrons about the offered produce? Do you have a system for documenting what customer purchase?
Other	Have you had any interactions with Country Fresh Stop administrators in which you had to decide between competing interests? (If yes) Can you describe that situation? How was it resolved? Is there anything else that you would like to share about your experience as a Country Fresh Stop?

**Table A2:** Donation Station Site Interview Guide.

Topic	Questions
Site Characteristics	What is your operation’s name? (What is it known by to those in the community?) What is the address of this location? Is it located in a church or other establishment? Is your operation sponsored by an organization (e.g., church, community organization)? Tell me a little about this pantry and how it works. For how many years has it been active? How frequently are you open to patrons? How many individuals work/volunteer at this location when the pantry is open? How many individuals do you serve during each distribution? What kinds of foods are distributed? How much produce (lb) do you typically distribute? What portion of it is fresh? What is your organization’s mission? What are some challenges that you face in pursuing this mission? (If active site) How does your Donation Station partnership help you to achieve that mission?
Program Participation and Perceptions	Did your site source produce from the Donation Station through Community Food Initiatives in 2019? (If yes) For how many years have you done so? (If no) For how many years did you do so? In what year did you stop? Tell me about your partnership with the Donation Station. Why did you originally choose to partner with the Donation Station? What appealed to you about the program? What has gone/went well? What are/were the challenges of being a Donation Station partner for your establishment? Is there anything that you would like to see changed about the program? (For current DS sites) Are there any factors that may impact your willingness to be a Donation Station partner moving forward? (For prior DS sites) Why did your site stop partnering with Donation Station? What resources or assistance would/would have help(ed) you to be a successful Donation Station site? How satisfied are/were you with the training, resources, and support provided to you as a Donation Station partner? How familiar are you with the produce auction from which most Donation Station produce is sourced (the Chesterhill Produce Auction)? How important was the source of the produce to you when considering your decision to participate as a Donation Station partner? Are you aware of how local farmer communities benefit from your organization through Donation Station? (If needed, explain the provenance of the produce purchased at auction and distributed to consumers.) Would this knowledge of benefitting local farmers affect how your organization operates at all? Why/why not?
Produce Inventory	Did you distribute produce to patrons prior to becoming a Donation Station site? (If yes) Did you offer any locally sourced produce? (If yes) Have you continued sourcing produce from anyone other than Community Food Initiatives? (If yes) Who/how/how often? What % is from CFI? (If no) Why did you stop sourcing from them? How often did/do you source produce from Community Food Initiatives? How many pounds of produce did/do you typically pick up on each occasion? What factors dictate how frequently you get produce (e.g., transportation)? What factors dictate how much produce you get at each pickup (e.g., storage)? Does the amount vary from distribution to distribution? What factors drive this variance (e.g., changing demand, changing capacity, etc.)? Are there certain types of produce that you prefer for your operation? (If yes) What types of produce do you prefer? *Show pictures, check off mentioned produce.* (If yes) How consistently are you able to get the produce that you prefer? (If yes) What factors drive your preference for certain types of produce? Is there any produce that patrons prefer consistently over others? Which one(s)? Are there any types of produce that patrons opt not to take? Which ones? How much produce (per person) do you provide to patrons at each distribution? Does it vary by distribution? Do you ever have produce left after a distribution? If so, how much (%)? How often? (If any) What do you do with produce that you are unable to distribute?
Distributions and Cost	Is the produce sourced from Community Food Initiatives labeled or marketed as being part of the Donation Station program? Is the Donation Station produce marketed any differently from other produce—such as canned or frozen—that is offered to patrons? (If any marketing) What costs do you incur in marketing your Donation Station produce to patrons? Probes: cost of materials? Of staff time? What strategies do you use to promote produce selection and consumption among your patrons (e.g., recipe tastings, recipe cards)? Do you have (additional) strategies for encouraging patrons to select the produce sourced from Community Food Initiatives (e.g., display, line order)?
Clientele	Could you tell me about the patrons of your site? Who is your target beneficiary? Do you see the same patrons frequently? What kinds of feedback have you received from patrons about the offered produce? Do you have a system for documenting the food received by each patron (e.g., amount, type, and/or value of food received)?
Other	Have you had any interactions with Donation Station administrators in which you had to decide between competing interests? (If yes) Can you describe that situation? How was it resolved? Is there anything else that you would like to share about your experience as a Donation Station partner?

## Appendix B

**Table A3:** Illustrative quotes from Country-Fresh-Stop- and Donation-Station-affiliated sites, organized by theme.

	Emergent Themes	Illustrative Quotes
Program Facilitators	Partner support	*“This last year they* [Rural Action] *provided the ordering, so I didn’t have to deal with that. Really made it easier this year … They provide signage and the displays … We did several different recipe cards, and they were provided at no cost to me.”* (CFS) *“… CFI staff are just so enthusiastic. Just, you know, if it can happen, they’ll make it happen. And if we can figure it out, they’re right there with you. It’s just a really … It’s just really great to work with them. And we’re all trying to reach the same goal. We’re all addressing food insecurity in our region. And they’re a big player. It’s just really wonderful. And maybe this once-a-month pantry that I kind of coordinate is a smaller player. But we just really appreciate everything they can do for us.”* (DS)
	Program stewardship	*“… I’m involved with working with our partners at Rural Action and just sort of getting things set up at the beginning of the year, and then our communications that go out to folks that want to receive information weekly about what we will have at the farm stand, because the items available changes weekly and we send an email to all of the people who have opted in to receive an email, so I manage that … And then we also have a gift shop manager at the [location], so what she does is enters the products that we’ll have each week into our sales system, so it’s an easy process for our consumers … they are buying that zucchini in the same way that they might buy a soda in our gift shop.”* (CFS) *“Before anybody can have anything off of that table, I take pictures and put it out on Facebook that it’s there. This is what’s here today. As soon as I’ve got it on Facebook, everybody’s welcome, and they’re waiting for me. I can show up at 10:00, and it maybe a half a dozen people. Some days there aren’t, but usually, there are people waiting for me … Yeah, it goes onto Facebook, and the librarian puts it out on Facebook, goes out on the village page, it goes out on my personal page.”* (DS)
Program Challenges	Seasonality	*“I do know patients said that they didn’t have many fruit options. And I would always respond with, “It’s seasonal. It’s hard. We’re getting this stuff locally.” And so a lot of the times they may not have it if it’s not in season. But they did seem to not have very much fruit options. But I think they sold out of fruit fairly quickly usually.”* (CFS) *“I think, I mean we really want to support that auction. We really want to support local farmers. When it works out that we can, we will. That will be the, always the option. In the winter when it’s not so available then it can’t. So, yeah.”* (DS)
	Food waste	*“I feel like I just lose a lot of money. But having said that, I had quality, fresh vegetables, people really liked it. I just feel that probably you need somebody who really knows how to utilize all of their vegetables doing it … [ACEnet partner] was really good with us because I would say what is going to last, and what can we do with it to get it to the next level. So, we would have [staff] cut things up, blanch them, freeze them, so that we could use it* (as part of site’s foodservice operation, if it did not sell). *But the same thing, we’d have cauliflower or broccoli, and it would go bad before we actually could get to it.”* (CFS) *“Also, depending on what we have leftover, sometimes we will take stuff over to the gathering place and they’re usually pretty happy to get that too. So really, really focused on trying to not waste the stuff and getting it to someone. It’s kind of silly to waste all this good food and just waste food in general, particularly given how food insecure this place is.”* (DS)
	CFI distribution	*“I’m not sure what traumatic words to use, but I think where there was a weariness of having to … There was such a competition and such an aggression and didn’t seem like there was a lot of structure. Like I don’t want to get involved with this.”* (DS)
Perceived Program Impacts	Support local	*“I think it’s important because it’s locally grown. It’s local people like us. We’re local. I don’t support big chain stores. I grew up here. I support mom and pops … And I hate going to Walmart. I hate going to them and supporting chains … You support mom and pops. Keep us in business as long as you can. I support Chesterhill Auction because they’re just we are. They’re trying to make a go of local farms, local dairies … ”* (CFS) *“I think also just the fresh aspect, the fact that it’s coming straight from farms and things like that. Still keeping things local and supporting people locally is important. It helps us get food to people in need and that’s kind of the point of the free meal programs.”* (DS)
	Patron satisfaction	*“And so, there were a few patients who if we didn’t have the Country Fresh Stop here, they would not have been able to purchase their own fruits and vegetables. They would have had to hire someone to go to the grocery store or ask a friend to go to the store for them. And they wouldn’t have been an active participant in I guess making those decisions on the foods they wanted to get. So, I really loved being able to see them go out and make a choice on some fruits and vegetables that they enjoyed eating … ”* (CFS) *“From conversations with our clients, they’re just really interested to hear that this was from the produce auction and raised locally. I think it’s an amount of pride … But I think it’s important to have some measure of pride in our region. And for a lot of folks, it’s a priority.”* (DS)
	Mission alignment	*“ … I do think having the ability to have food, fresh food and a Country Fresh Stop nearby, it takes away some of the difficulty in having to travel to get fresh produce as well as if it’s reinforced in say a doctor visit here. To say, “Hey, this is one way that you can make a change to your health, eat more fruits and vegetables.” And they go outside and they see that there’s the ability to do that, and they can see that the prices are not too bad … So, I do think having fresh food on hand right nearby, it makes it easier for them to make that decision without having to consider too many other things.”* (Pop-up market at healthcare site, CFS) *“Hungry kids can’t learn. If you’re feeding kids, they will learn. That’s the school’s mission. Its job is to educate kids. But at the same time, they understand that they can’t educate hungry kids. Everything I bring in there helps resolve the hungry kid issue. Same thing at the library in a lot of ways. They’re a place of education, and you’re educating the whole person. If there’s food there for them, that helps them. It’s not an officially stated goal, it’s just understood, it’s hungry people, so you feed them. As I said before, the library is very much a community center in that little village, and it’s there, it’s where people come. That’s where we take the food.”* (DS)
